# Phase separation liquid-liquid extraction for the quantification of 8-iso-Prostaglandin F2 Alpha in human plasma by LC-MS/MS

**DOI:** 10.5937/jomb0-24746

**Published:** 2021-01-26

**Authors:** Desislav G. Tomov, Georgeta Bocheva, Vidka Divarova, Lilia Kasabova, Dobrin Svinarov

**Affiliations:** 1 Technological Center for Emergency Medicine, Plovdiv, Bulgaria; 2 Medical University of Plovdiv, Faculty of Pharmacy, Department of Bioorganic Chemistry, Plovdiv, Bulgaria; 3 Medical University of Sofia, Faculty of Medicine, Department of Pharmacology and Toxicology, Sofia, Bulgaria; 4 Medical University of Plovdiv, Faculty of Pharmacy, Department of Chemical Sciences, Plovdiv, Bulgaria; 5 Medical University of Sofia, Faculty of Medicine, UMBAL Alexandrovska, Clinical Laboratory & Clinical Pharmacology, Sofia, Bulgaria

**Keywords:** 8-iso-Prostaglandin F2a, liquid extraction with phase separation, LC-MS/MS, LC-MS/MS, ekstrakcija tečnosti sa odvajanjem faza, 8-izo-prostaglandin F2a

## Abstract

**Background:**

Reactive oxygen species (ROS) are produced in the body during normal metabolism by means of enzymes and non-enzymatic chemical reduction of molecular oxygen. In case of the prevalence of ROS formation over their elimination, highly reactive free radicals can be accumulated and can cause multiple damages to the biomolecules and cells. Determination of isoprostanes in biological matrices is most often used to register free radical damage and requires selective, sensitive and specific techniques.

**Methods:**

This study presents the development and validation of the LC-MS/MS method for the determination of 8-iso-Prostaglandin F2α in human plasma utilising a modified liquid-liquid extraction procedure with phase separation.

**Results:**

Modified sample preparation procedure assured higher extraction yield, clear separation of organic layer from the plasma water phase and protein precipitates, and better-purified product for instrumental analysis. Linearity was validated in the range 0.1-5.0 µg/L with R2 > 0.996; normalised matrix varied between 86.0% and 108.3%, accuracy ranged from 90.4 % to 113.9% and precision both within runs and between runs was less than 7%. With a run time of 10 min, a throughput of over 50 samples per working day could be performed.

**Conclusions:**

The method meets all the current industrial validation criteria and allows the accurate and precise determination of 8-iso-PGF2α in human plasma at diagnostically significant concentration range.

## Abstract (sr)

**Uvod:**

Reaktivne vrste kiseonika (ROS) nastaju u telu tokom normalnog metabolizma pomoću enzima i neenzimske hemijske redukcije molekularnog kiseonika. U slučaju bržeg formiranja ROS od njihovog eliminisanja, mogu se akumulirati visoko reaktivni slobodni radikali i mogu izazvati višestruka oštećenja biomolekula i ćelija. Određivanje izoprostana u biološkim matricama najčešće se koristi za registrovanje oštećenja prouzrokovana slobodnim radikalima i zahteva selektivne, osetljive i posebne metode rada.

**Metode:**

Ovo istraživanje prikazuje razvoj i validaciju LC-MS/MS metode za određivanje 8-izo-prostaglandina F2α u ljudskoj plazmi pomoću modifikovanog postupka ekstrakcije tečno-tečno sa razdvajanjem faza.

**Rezultati:**

Modifikovana procedura pripreme uzoraka osigurala je veći učinak ekstrakcije, jasno odvajanje organskog sloja od vodene faze u plazmi i taloga proteina, i bolje pročišćen proizvod za instrumentalnu analizu. Linearnost je potvrđena u opsegu 0,1-5,0 µg/L sa R2 > 0,996; normalizovana matrica varirala je između 86,0 i 108,3%, tačnost je bila u rasponu od 90,4% i 113,9%, a preciznost i u toku i između faza bila je manja od 7%. U toku radnog dana može se izvršiti propusnost od preko 50 uzoraka sa trajanjem od 10 minuta.

**Zaključak:**

Ova metoda ispunjava sve trenutne kriterijume industrijske validacije i omogućava tačno i precizno određivanje 8-izo-PGF2α u ljudskoj plazmi u dijagnostički značajnom rasponu koncentracije.

## Introduction

Reactive oxygen species (ROS) are produced in the body during normal metabolism both by means of enzymes and non-enzymatic chemical reduction of molecular oxygen (O_2_). In case of the prevalence of ROS formation over their elimination, highly reactive free radicals can be accumulated and can alter cell functions by causing multiple damages to the biomolecules, including proteins, DNA and lipids, such as polyunsaturated fatty acids (PUFAs) [1] [2] [3]. The imbalance caused by increased free radicals accumulation and/or decreased antioxidant defence is considered as oxidative stress (OS). ROS are short-lived and are difficult to assess both *in vitro* and *in vivo*. Thus, measurement of more stable oxidation products left by ROS in biological fluids and tissues is a well-accepted and widely-applied method for the quantification of OS *in vivo* [4] [5] [6]. The particular reaction of ROS with lipids is known as »lipid peroxidation,« which is one of the major mechanisms leading to cellular damage and ultimately to cell death [1] [7] [8] [9]. The prominent products generated by lipid peroxidation are malondialdehyde and oxidised arachidonic acid derivatives, like F_2_ isoprostanes [10] [11] and 15(S)-8-iso-PGF_2α_ in particular, which belong to the most studied OS biomarkers [12] [13] [14].

Unlike other lipid peroxidation products (e.g. lipoperoxides and aldehydes), isoprostanes are less reactive and relatively stable [4] [15]. Isoprostanes can be found in almost all biological fluids including blood plasma, urine [16] and cerebrospinal fluid [17], and their concentration in the tissues, in specific body fluids, or in condensate from exhaled air [18] [19], can provide information about OS in a particular organ or system [20]. Endogenous isoprostanes are found at low concentrations in biological matrices [21] and either the methods for simultaneous determination of several of them or methods for the measurement of single isoprostanes are employed for the assessment of OS. The major single isoprostane is 15(S)-8-iso-PGF_2α_, and its quantification is considered as one of the most reliable approaches for the assessment of ROS damage [12] [13] [14]. The determination of isoprostanes in biological matrices requires selective, sensitive and specific techniques, and despite the variety of published methods, there are still unresolved analytical challenges. The major difficulties are related to their oxidation or their artificial ex vivo formation [22], as well as their low quantities in the samples.

The quantification of isoprostanes employs radio immune assays (RIA), enzyme-linked immune-sorbent assays (ELISA), gas chromatography (GC), high-performance liquid chromatography (HPLC, LC) and capillary electrophoresis with conventional (UV, fluorescence) and mass spectrometric detection. These techniques were recently reviewed by Thakare et al. [23]. All of them have advantages, disadvantages, and limitations [16] [21] [24] [25]: lack of sufficient selectivity and specificity (ELISA and RIA), extensive sample preparation and chemical derivatisation (GC) etc. Liquid chromatography with tandem mass spectrometry (LC-MS/MS) is the preferred technique for eicosanoid detection in the last 10-15 years, since it provides improved specificity and selectivity, ultimate sensitivity, and much easier sample pre-treatment, compared to GC [26] [27] [28] [29]. Efficient sample preparation is a critical first step of all separation methods used for the quantification of isoprostanes in biological matrices.

Sample pre-treatment procedures include protein precipitation, liquid-liquid extraction (LLE) and solid-phase extraction with numerous modification steps aiming to increase extraction recovery and decrease matrix effect, such as liquid phase microextraction, single drop micro-extraction, dispersive liquid-liquid micro-extraction, supported liquid membranes, electro membrane extraction, supported liquid extraction, and others [4]. Despite the plethora of methods and sample preparation approaches employed for analysis of isoprostanes, certain advantages and disadvantages, common analytical challenges and unresolved problems still remain to be addressed.

The aim of this study was to develop and validate sensitive and selective determination of 15(S)-8iso-PGF_2α_ in human plasma by LC-MS/MS, utilising classic LLE, improved by phase separation.

## Material and Methods

### Chemicals and reagents

The standards of 8-iso-PGF_2α_ and its deuterated analogue (8-iso-PGF_2α_-d4) were purchased from Cayman Chemicals (Ann Arbor, MI, USA); LC-MS-grade methanol (MeOH) was delivered by VWR Chemicals BDH (Avantor, USA); 98% formic acid, ethyl ethanoate, hexane, methylene chloride, and sodium phosphate monobasic were obtained from Sigma-Aldrich (St. Louis, MI, USA). Stock solutions of 8-iso-PGF_2α_ (1.0 mg/L), of 8-iso-PGF_2α_-d4 (1.0 mg/L) and working internal standard solution of 8iso-PGF_2α_-d4 (10 mg/L) were prepared in 50% MeOH. From separately prepared stocks, working solutions of 8-iso-PGF_2α_ were prepared in 50% MeOH with concentrations of 10, 16, 20, 50, 100, 200 and 500 µg/L for the preparation of the calibration curve (CC) samples, and with concentrations of 10, 25, 250, and 400 µg/L for the preparation of the lower limit of quantification (LLOQ) and quality control (QC) samples at three levels.

### Preparation of calibration curve (CC) and quality control (QC) samples

Plasma from young, healthy subjects who consented to participate in the study, immediately separated from blood cells at 4 °C after blood withdrawal and pooled promptly, was used for the preparation of CC and QC samples as follows: to 1980 µL of plasma pool 20 µL, of the respective CC and LLOQ/QC, working solutions were added, mixed gently for 5 min, and frozen at -20 °C, CC concentrations being 0.10, 0.16, 0.20, 0.50, 1.0, 2.0 and 5.0 µg/L; LLOQ/QC I -III levels -0.1/0.25, 2.5 and 4.0 µg/L.

### Sample preparation procedure

In the development of the method, we employed a modified liquid-liquid extraction utilising phase separation. The procedure consists of eight consecutive steps: to a 15 mL sample tube, 500 µL human plasma (CC, QC or patient-derived), and 100 µL of internal standard solution (IS) were added and gently mixed by vortex for 1 min; further, 500 µL of pre-saturated NaH_2_PO_4_ solution and 4.0 mL of ethyl ethanoate were added, and the sample was intensively mixed by vortex for 6 min; after centrifugation for 10 min at 2500 g, three distinct layers were obtained -upper organic and lower aqueous, which were separated by a layer of precipitated and salted out plasma proteins. The upper organic layer was transferred to another tube and evaporated under a gentle stream of nitrogen at 40 °C. The dry residue was redissolved in 100 µL of MeOH-water (1:1; v/v) and injected for analysis.

### Liquid chromatographic and mass spectrometric conditions

The instrument consisted of Ultimate 3000 LC system equipped with a quaternary pump, an autosampler and a thermostat for chromatographic columns, and TSQ Quantum Access Max triple quadru pole mass spectrometer (Thermo Fisher Scientific, MA, USA). Chromatographic separation was performed under isocratic conditions on a coreshell Accucore^TM^ RP-MS 100 x 2.1 mm, 2.6 mm particles analytical column (Thermo Fisher Scientific, MA, USA), with a mobile phase consisting of 0.1% formic acid in MeOH-water (65:35; v/v), flow rate 0.25 mL/min.

Heated electrospray ionisation (HESI) was used for analyte detection in negative ionisation mode with spray voltage -4000 V; source temperature 400 °C; sheath gas, 45 arbitrary units; vaporiser temperature 280°C; capillary temperature 300 °C. Deprotonated molecules of analyte and IS were used as precursor ions for selected reaction monitoring (SRM) with transitions of m/z 353.2 → 193.1 for 8-iso-PGF_2α_ and 357.2 → 197.2 for 8-iso-PGF_2α_-d4.

Argon was used as collision gas; collision energy was 28 V. Calculation of concentrations was performed by the method of background subtraction.

### Method validation

Selectivity was assessed with nine individual matrices of human plasma, including lipemic, hemolytic and icteric, applying the technique of standard additions at two concentration levels with predefined normalised matrix effect within 85-115%. Imprecision and inaccuracy should also be in the range of 15% within and between runs for QC samples, and within ±20% for the LLOQ sample; linearity in the defined CC range, with R2>0.996. Freezethaw stability should be verified for three cycles each lasting 24 h, post-preparative stability for ten h at 4°C , short term stability of working solutions at room temperature for 72 hours at daylight and for 72 hours in the dark, stock solution stability and long term stability in plasma for over three months at -20 °C; all of the above within 15% of theoretical. Validation experiments were designed according to current EMA/FDA industrial guidance for bioanalysis via LC-MS/MS [30] [31], encompassing four consecutive analytical runs, performed in four consecutive working days for the assessment of precision and accuracy, each with separate CC, with five replicates of the LLOQ and QC samples in the first day, and duplicate analysis of the LLOQ and QC samples in the next three days. A separate set of experiments was performed for validation of selectivity, matrix effect and stability of the method.

## Results and Discussion

Although serum and plasma are considered similar, immediately separated plasma samples are typically used for eicosanoid and isoprostane measurements to avoid their oxidation and the clotting reaction in serum that leads to artificial in vitro increase of their concentrations. Protein molecules have different solubility in water, based on the amount and the type of amino acid residues on their surface. In aqueous solution, proteins have a coating of water molecules that stabilise them and prevent their aggregation. In our method for sample preparation, by employing the salting-out effect of saturated NaH_2_PO_4_, water envelope of plasma proteins is disturbed, and efficient protein precipitation takes place, which enhances extraction yield, purification and facilitates the separation of clear organic layer for further processing. However, in order to obtain complete precipitation of the proteins in a biological sample, it is necessary to fully saturate the solution, which can be achieved by using plenty of dry substance or appropriate volume of the pre-saturated solution of the given salt. In our sample pre-treatment procedure, pre-saturated NaH_2_PO_4_ was used to precipitate the proteins and ethyl ethanoate to extract both the analyte 8-iso-PGF_2α_ and IS 8-iso-PGF_2α_-d4.

**Figure 1:**
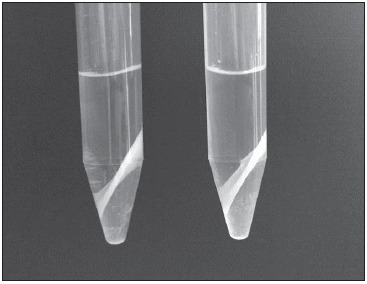
Sample tubes after centrifugation with clearly visible protein precipitate in the middle, separating the organic upper layer from the lower plasma water layer

Figure 1 presents a picture of the sample after centrifugation with stable, thick and clearly visible protein precipitate separating the organic upper layer from the lower plasma water layer. Comparative experiment with the addition of an equal volume of water instead of the pre-saturated solution of NaH_2_PO_4_ showed 5-fold lower recovery for both analytes (Table 1).

**Table 1:** Comparison between the recovery with and without saturated NaH_2_PO_4_ * – 1 and 2 for duplicate extractions; H_2_O – for using an equal volume of water instead of saturated (s) NaH_2_PO_4_

Sample name	Recovery 8-iso-PGF2a	Recovery 8-iso-PGF2a-d4
H_2_O_1*	9.60%	8.80%
H_2_O_2	11.80%	8.50%
s NaH_2_PO_4__1	59.20%	49.20%
s NaH_2_PO_4__2	68.50%	51.60%

The above sample pre-treatment is a modification of a more general procedure developed in our laboratories entitled phase separation protein precipitation, in which serum or plasma are treated with MeOH, ethanol or acetonitrile, organic solvents fully miscible with water, in the presence of saturated salt solution. In this protein precipitation method, instead of obtaining two layers after centrifugation – supernatant as a mixture of plasma water and respective organic solvent, and precipitate on the bottom of the tube, three clearly separated layers are achieved as shown in Figure 1 – upper organic phase, protein precipitate in the middle, and lower plasma water phase. Thus, simplicity of protein precipitation is combined with extraction and purification – components of interest could be predominantly ether in the organic layer, or in the plasma water phase, both of which could be further used for analysis.

**Figure 2:**
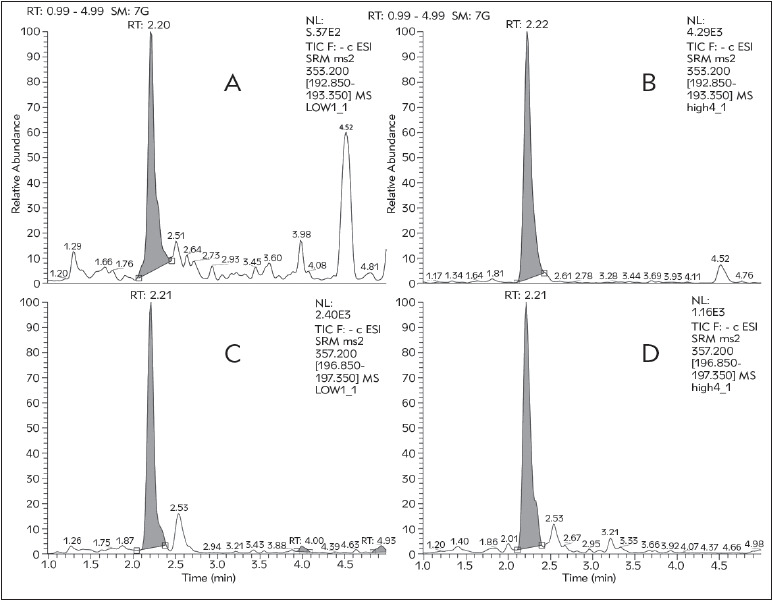
Chromatograms obtained from QC sample analysis. Concentrations of 8-iso-PGF2α are: 0.25 µg/L (A) and 4.0 µg/L (B), with the internal standard under each (C, D)

**Figure 3:**
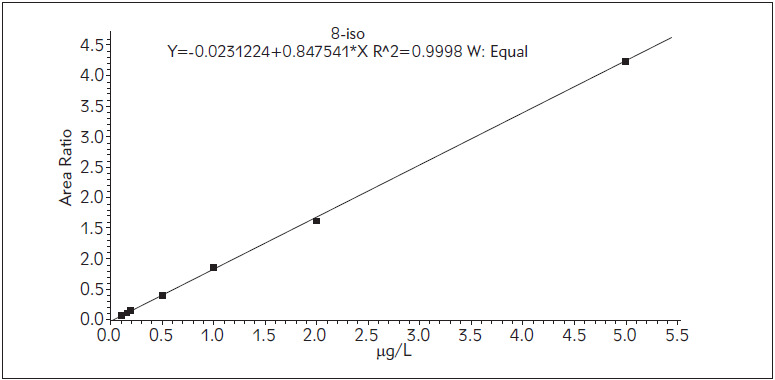
Calibration curve of 8-iso-PGF_2α_

SRM chromatograms for the QC samples, obtained according to the described method are presented in Figure 2. Linearity was assured in the predefined range 0.1-5.0 µg/L with R2=0.9998 and excellent linear equation (Figure 3). Accuracy and precision calculated from the LLOQ and QC samples fully met the pre-defined acceptance criteria (Table 2).

**Table 2:** Accuracy and precision of the assay

Level	Accuracy (% from theoretical)				Precision (CV %)	
	within-run (n=5)		between-run (n=3)		within-run (n=5)	between-run (n=3)
	min	max	min	max		
LLOQ (0.1 mg/L)	90.4%	104.0%	90.4%	113.9%	5.7%	6.9%
QC I (0.25 mg/L)	91.4%	105.2%	87.6%	105.2%	5.6%	6.2%
QC II (2.5 mg/L)	100.2%	111.8%	91.6%	111.8%	4.2%	6.7%
QC III (4.0 mg/L)	105.1%	109.4%	94.8%	109.4%	1.5%	5.4%

Normalised matrix effect was also fully acceptable, being in the range 86.0%-106.5%, except for a single plasma sample with excessive haemolysis, where normalised matrix effect reached 123.1%. It should be noted that significant ion suppression found for the individual plasma samples was efficiently compensated by the stable isotope labelled internal standard (Table 3).

**Table 3:** Matrix effect of the assay, performed with 18 plasma matrices *: VI_SA1 – VI_SA12: 12 individual plasma matrices, including 2 icteric (VI_SAB1 and VI_SAB2), 2 haemolytic (VI_SAH1 and VI_SAH2) and 2 lipemic (VI-SAL1 and VI_SAL2) ones

Sample*	Matrix effect 8-isoPGF2a	Matrix effect 8-isoPGF2a-D4	Normalised matrix effect
VI_SA1	26.2%	30.4%	86.0%
VI_SA2	51.7%	53.0%	97.5%
VI_SA3	59.2%	62.9%	94.1%
VI_SA4	62.9%	60.9%	103.2%
VI_SA5	60.3%	57.8%	104.3%
VI_SA6	59.1%	56.0%	105.5%
VI_SA7	48.0%	53.0%	90.7%
VI_SA8	45.0%	48.4%	93.1%
VI_SA9	42.5%	42.4%	100.3%
VI_SA10	38.7%	40.9%	94.7%
VI_SA11	45.1%	45.9%	98.3%
VI_SA12	43.6%	43.5%	100.2%
	** min 26.2% **	** min 30.4% **	** min 86.0% **
	** max 62.9% **	** max 62.9% **	** max 105.5% **
VI_SAB1	60.3%	56.6%	106.5%
VI_SAB2	44.3%	47.3%	93.7%
VI_SAH1	82.7%	67.1%	123.1%
VI_SAH2	47.6%	47.7%	99.8%
VI_SAL1	57.6%	54.2%	106.2%
VI_SAL2	51.1%	51.2%	99.8%

Stability of working solutions estimated at room temperature and at 4-8 °C, was between -2% and ±6.9%, well within the pre-defined criteria. Freezethaw stability for three cycles of 24 hours each, was between -1.9% and +5.1%, also within the predefined criteria.

Some limitations of our method include the observed ion suppression effect, which though is fully compensated by the use of stable isotope-labelled internal standard, and LLOQ at the commonly accepted cut-off limit for registration of free radical damage. We applied the above sample pre-treatment for analysis with a more sensitive instrument and achieved significantly better sensitivity (unpublished data).

## Conclusions

This study presents an improved method for the determination of 8-iso-PGF_2α_ by LC-MS/MS in human plasma utilising modified LLE with phase separation. The proposed procedure is easy to implement and assures higher extraction yield, clear separation of organic layer from the plasma water phase and protein precipitates, and better-purified product for instrumental analysis. HPLC separation was optimised with the use of a C18 core-shell column and triple quadrupole MS/MS analysis provided the required selectivity and specificity. With a run time of 10 min, a throughput of over 50 samples per working day could be performed. The method was validated according to the current industrial requirements and allowed for the accurate and precise determination of 8-iso-PGF_2α_ in human plasma at diagnostically significant concentration range.

## Conflict of interest statement

The authors stated that they have no conflicts of interest regarding the publication of this article.

## List of abbreviations

CC, calibration curve; GC, gas chromatography; HPLC, LC, liquid chromatography; IS, internal standard; MeOH, methanol; OS, oxidative stress; QC, quality control; ROS, reactive oxygen species; SRM, selected reaction monitoring.
